# Study on the CBN Wheel Wear Mechanism of Longitudinal-Torsional Ultrasonic-Assisted Grinding Applied to TC4 Titanium Alloy

**DOI:** 10.3390/mi13091480

**Published:** 2022-09-06

**Authors:** Junli Liu, Zhongpeng Liu, Yanyan Yan, Xiaoxu Wang

**Affiliations:** School of Mechanical and Power Engineering, Henan Polytechnic University, Jiaozuo 454003, China

**Keywords:** TC4 titanium alloy, longitudinal-torsional ultrasonic grinding, grinding force, grinding temperature, grinding wheel wear

## Abstract

In this study, the CBN (cubic boron nitride) wheel wear model of TC4 titanium alloy in longitudinal-torsional ultrasonic-assisted grinding (LTUAG) was established to explore the grinding wheel wear pattern of TC4 titanium alloy in LTUAG and to improve the grinding efficiency of TC4 titanium alloy and the grinding wheel life. The establishment of the model is based on the grinding force model, the abrasive surface temperature model, the abrasive wear model, and the adhesion wear model of TC4 titanium alloy in LTUAG. The accuracy of the built model is verified by the wheel wear test of TC4 titanium alloy in LTUAG. Research has shown that the grinding force and grinding temperature in LTUAG increase with the increase of the grinding depth and workpiece feed rate and decrease with the increase of the longitudinal ultrasonic amplitude. It also shows that the grinding force gradually decreases with the increase of the grinding wheel speed, while the grinding temperature gradually increases with the increase of the grinding wheel speed. In addition, the use of LTUAG can significantly reduce the wear rate of the grinding wheel by 25.2%. It can also effectively reduce the grinding force and grinding temperature.

## 1. Introduction

Since its inception in the middle of the last century, titanium alloy has been used as an ideal material for key spacecraft components such as space shuttle skins and compressor blades because of its excellent physical properties, such as corrosion resistance, heat resistance, fatigue resistance, and high specific strength [1,2,3,4]. However, the hardness and strength of titanium alloy are high, and the coefficient of thermal conductivity is poor, so the cutting performance is poor. The large cutting force and cutting temperature also lead to the poor surface quality of the workpiece, and the tool wear is more serious, which affects the promotion and application of titanium alloy material. In recent years, the ultrasonic-assisted grinding technology developed by combining conventional grinding technology and ultrasonic vibration processing technology has exhibited characteristics such as instantaneous effect, intermittent contact, high-frequency cutting, and so on, which have a positive effect on reducing the grinding force, grinding heat, and wheel wear. Therefore, ultra-precision machining of titanium alloy material can be achieved [5,6,7]. At the same time, scholars have also conducted related research on the wear of grinding wheels under longitudinal-torsional ultrasonic-assisted grinding (LTUAG) of titanium alloys.

Through a large number of experimental studies, Bhushan [8] classified grinding wheel wear types into mechanical wear, chemical wear, and diffusion wear. Among them, the typical forms of mechanical wear include abrasive wear, plastic wear, and thermal stress fracture. Churi et al. [9] studied the grinding wheel wear mechanism of Ti6Al4V titanium alloy during LTUAG, and the study showed that the diamond grinding wheel wear form of longitudinal ultrasonic vibration grinding of Ti6Al4V titanium alloy is abrasive wear, abrasive particle shedding, abrasive particle crushing, and binder fracture. Abrasive grains on the edge of the end face of the grinding wheel are more obviously broken and fall off after grinding, and abrasive particles near the center of the wheel end face are less worn.

Qin [10] established a tool wear model for titanium alloy in rotary ultrasonic grinding based on dimensional analysis, which reflects the influence of tool structure parameters, grinding process parameters, and ultrasonic parameters on tool wear. The validity and significance test of the model found that the F distribution value of the model was 45.04, which was much higher than the critical value of the F distribution, indicating that the established tool wear model had a high degree of significance and could predict tool wear to a certain extent. Liu [11] established a finite element model for multi-abrasive rotary ultrasonic grinding of Ti6Al4V titanium alloy, and on this basis, the secondary development of the wear subprogram of Deform software was carried out. Thus, the finite element simulation of the model was realized. The research results show that the wear amount of the grinding wheel under the rotating ultrasonic grinding of Ti6Al4V titanium alloy decreases with the increase of the abrasive particle size and grinding wheel speed and increases with the increase of the abrasive concentration, workpiece feed rate, and ultrasonic frequency. The finite element simulation results are consistent with the variation pattern of the experimental results, thus proving the correctness of the finite element model. Li et al. [12] conducted ordinary grinding and ultrasonic-assisted grinding tests for TC4 titanium alloy. The study found that compared to ordinary grinding, the abrasive debris adhering to the surface of the workpiece after ultrasonic-assisted grinding was significantly reduced, which proved that ultrasonic-assisted grinding can effectively reduce the abrasive debris adhesion phenomenon in the process of titanium alloy grinding and can improve the adhesion and blockage of the grinding wheel surface, thereby improving the surface quality of the TC4 titanium alloy.

To sum up, there is little research on the wheel wear mechanism of titanium alloy ultrasonic-assisted grinding by scholars, and the related research mainly focuses on the grinding wheel wear mechanism under the condition of one-dimensional ultrasonic-assisted grinding, where vibration is applied to the grinding wheel or workpiece. The grinding wheel wear mechanism under the LTUAG of titanium alloy is rarely involved. From this background, TC4 titanium alloy was chosen as the research object, and the LTUAG method was adopted. The grinding wheel wear during LTUAG of TC4 titanium alloy is studied from the perspective of theoretical modeling and experimental research, which is expected to provide a theoretical reference for the high-efficiency precision grinding of TC4 titanium alloy by longitudinal-torsion ultrasonic-assisted grinding.

## 2. Establishment of a Wheel Wear Model of TC4 Titanium Alloy in LTUAG

### 2.1. Analysis of Kinematics Characteristics of TC4 Titanium Alloy in LTUAG

**Figure 1:**
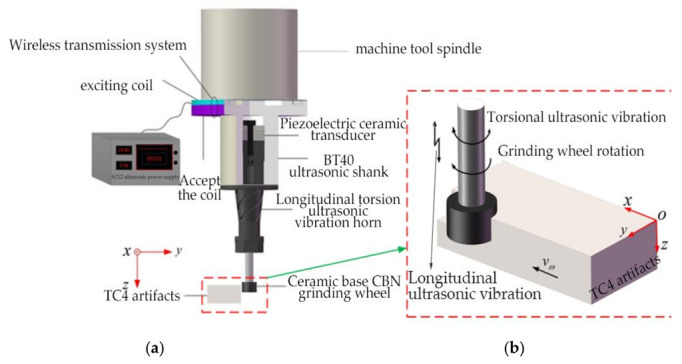
Schematic diagram of the longitudinal-torsional ultrasonic-assisted grinding (LTUAG) system and its motion model. (**a**) Schematic diagram of the grinding system in LTUAG. (**b**) Schematic diagram of the motion model in LTUAG.

The LTUAG system is shown in Figure 1a, which is mainly composed of a longitudinal-torsional ultrasonic vibration system and a ceramic-based CBN (cubic boron nitride) grinding wheel. The longitudinal-torsional ultrasonic vibration system is composed of an ultrasonic generator (working frequency range: 20–50 kHz), a wireless transmission system, a BT40 ultrasonic shank (Huizhuan Technology Group Co., Ltd., Guangzhou, China), a piezoelectric ceramic transducer, and a longitudinal-torsion ultrasonic vibration amplitude transformer. According to Figure 1b, and assuming that the workpiece is stationary relative to the grinding wheel, the movement of the abrasive particles on the surface of the grinding wheel during LTUAG includes the feed movement along the *x*-axis with a speed of *v_w_*, the motion work in the *xoy* plane includes rotational motion around the *z*-axis with a rotational speed of *n*, a torsional ultrasonic vibration with an amplitude of *b*, and a longitudinal ultrasonic vibration with an amplitude of *a* along the *z*-axis.

To facilitate the analysis of the kinematic characteristics of abrasive particles, it is assumed that the workpiece material is uniform and isotropic, the abrasive particles on the surface of the grinding wheel are uniformly distributed in the longitudinal and axial directions, and the frequency and output amplitude of the longitudinal-torsional ultrasonic vibration system remain stable during the machining process. According to the coordinate system shown in Figure 1b, the motion of a single abrasive particle during LTUAG can be expressed as:

(1) $$\{\begin{array}{l} {x(t)=v}_{w}\cdot t+R\cdot sin\left[\omega tb\cdot {sin(2\pi f}_{b}t+\varphi )/R\right]\\ y(t)=R\cdot cos\left[\omega t+b\cdot {sin(2\pi f}_{b}t+\varphi )/R\right]\\ {z(t)=asin(2\pi f}_{a}t) \end{array}$$

where *v_w_* is the workpiece feed speed, *R* is the grinding wheel radius, ω is the grinding wheel angular speed, *b* is the torsional ultrasonic vibration amplitude, *a* is the longitudinal ultrasonic vibration amplitude, *f_b_* is the torsional-ultrasonic vibration frequency, *f_a_* is the longitudinal ultrasonic vibration frequency, and *φ* is the phase difference between longitudinal and torsional vibration.

According to Equation (1), taking the derivative of time, the velocity of a single abrasive particle in LTUAG can be obtained as:

(2) $$\{\begin{array}{l} v_{x}{=x\prime }^{}{(t)=v}_{w}+\left[\omega R+2\pi fbcos(2\pi ft+\varphi )\right]cos\left[\omega t+\frac{b}{R}sin(2\pi ft+\varphi )\right]\\ v_{y}{=y\prime }^{}(t)=-\left[\omega R2\pi fbcos(2\pi ft+\varphi )\right]sin\left[\omega t+\frac{b}{R}sin(2\pi ft+\varphi )\right]\\ v_{z}{=z\prime }^{}(t)=2\pi fcos(2\pi ft) \end{array}$$

According to Equation (2), the grinding arc length, *l_c_*, of a single abrasive particle in LTUAG in one vibration cycle can be obtained as:

(3) $$\begin{array}{ll} l_{c} & =\int_{0}^{t}\sqrt{v_{x}^{2}{+v}_{y}^{2}{+v}_{z}^{2}}dt\\ & =\int_{0}^{t}\sqrt{\begin{array}{c} v_{w}^{2}{+2v}_{w}cos\left[\omega t+\frac{b}{R}sin(2\pi ft+\varphi )\right]\left[\omega R+2\pi fbcos(2\pi ft+\varphi )\right]+\\ {\left[\omega R+2\pi fbcos(2\pi ft+\varphi )\right]}^{2}+{\left(2\pi fa\right)}^{2}{cos}^{2}\left(2\pi ft\right) \end{array}}dt \end{array}$$

**Figure 2:**
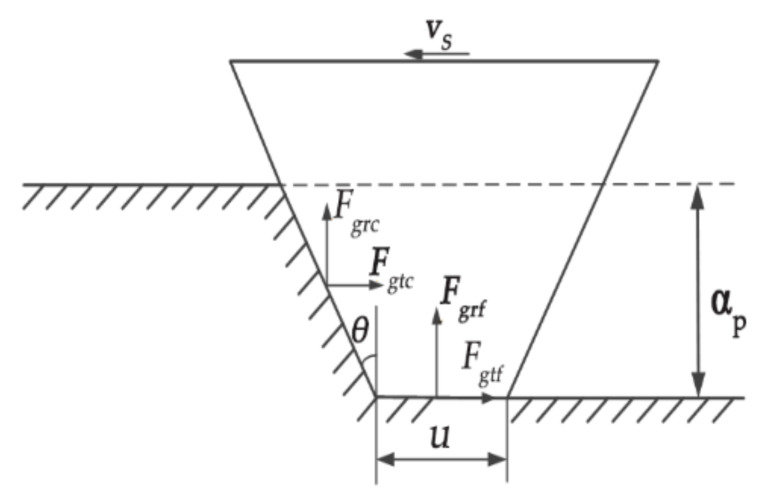
Grinding force model of a single CBN abrasive particle in LTUAG. (In the figure: *v_s_* is the grinding linear speed, *a_p_* is the grinding depth of a single abrasive particle in LTUAG, *F_gt_*_c_ is the tangential deformation force for wear debris of a single abrasive particle, *F_gtf_* is the tangential friction of a single abrasive particle, *F_grc_* is the radial deformation force for wear debris of a single abrasive particle, *F_grf_* is the radial friction of a single abrasive particle.).

When *a* = *b* = 0, according to Equation (3), the ordinary grinding arc length, *l_p_*, of a single abrasive particle can be expressed as:

(4) $$l_{p}=\int_{0}^{t}\sqrt{v_{w}{}^{2}{+v}_{a}^{2}{+2v}_{w}v_{a}cos(\omega t)}dt$$

where *v_a_* is the rotational speed of the grinding wheel. According to Figure 2 and Equation (4), the removal volume, *V_p_*, of a single abrasive grain material during ordinary grinding can be obtained as:

(5) $$V_{p}{=S}_{p}l_{p}=\frac{1}{2}{(2u+2a}_{p}{tan\theta )a}_{p}\int_{0}^{t}\sqrt{v_{w}{}^{2}{+v}_{a}^{2}{+2v}_{w}v_{a}cos(\omega t)}\mathrm{dt}$$

where *S_p_* is the cross-sectional area of ordinary grinding chips.

It can be seen from Equation (3) that the arc length of the single abrasive particle in LTUAG is related to the workpiece feed rate, the linear speed of the grinding wheel, the longitudinal torsional ultrasonic vibration frequency, the longitudinal ultrasonic vibration amplitude, and the torsional ultrasonic vibration amplitude. It can be seen from Equations (3)–(5) that, under the same grinding conditions, the arc length of the single abrasive particle in LTUAG is longer than that in ordinary grinding. This means that LTUAG introduced changes to the grinding mechanism of the single abrasive particle and improved the time for the single abrasive particle to participate in grinding. Therefore, it is beneficial to improve the material removal rate of LTUAG.

### 2.2. Establishment of a Grinding Force Model for Titanium Alloy in LTUAG

The grinding force and grinding heat generated in LTUAG increase the mechanical stress and thermal stress acting on the surface of the abrasive particle, which exceeds the strength of the abrasive particle and the binding agent itself, which causes the breaking and shedding of abrasive grains, eventually leading to grinding wheel wear. Therefore, the study of the grinding force of titanium alloy in LTUAG is of great significance for the subsequent establishment of the grinding wheel wear model of the titanium alloy in LTUAG. In the study, a single CBN abrasive particle was simplified as a flat-ended quadrangular pyramid. The cross-sectional shape of the abrasive grain can be approximated as an isosceles trapezoid. According to the cross-sectional shape of CBN abrasive grains and the force in LTUAG, the grinding force model of a single CBN abrasive particle in LTUAG was established, as shown in Figure 2.

As shown in Figure 2, the grinding force is mainly composed of two parts: one is the tangential and radial deformation resistance of the abrasive particles acting on the rake face of the abrasive grain, and the other is the tangential and radial friction force between the bottom surface of the abrasive grain and the workpiece surface. The tangential grinding force, *F_gt_*, and the radial grinding force, *F_gr_*, of a single abrasive particle in LTUAG can be expressed as:

(6) $$\{\begin{array}{c} F_{\mathrm{gt}}{=F}_{gtc}{+F}_{gtf}\\ F_{gr}{=F}_{grc}{+F}_{grf} \end{array}$$

where *F_gt_*_c_ is the tangential deformation force for wear debris of a single abrasive particle, *F_gtf_* is the tangential friction of a single abrasive particle, *F_grc_* is the radial deformation force for wear debris of a single abrasive particle, and *F_grf_* is the radial friction of a single abrasive particle. According to the research of Yan et al. [13], the tangential grinding force, *F_gt_,* and radial grinding force, *F_gr_*, of a single abrasive particle in LTUAG can be expressed as:

(7) $$\{\begin{array}{l} F_{gt}{=F}_{gtc}{+F}_{gtf}=\frac{{\pi F}_{0}a_{p}^{2r}{tan}^{r-1}\theta \cdot \left({u+a}_{p}tan\theta \right)a_{p}\int_{0}^{t}\sqrt{v_{w}{}^{2}{+v}_{a}^{2}{+2v}_{w}v_{a}cos(\omega t)}dt}{4\int_{0}^{t}\sqrt{v_{w}^{2}{+v}_{s}{}^{2}{+2v}_{w}v_{s}cos\left[\omega t+\frac{b}{R}sin(2\pi ft+\varphi )\right]+{\left(2\pi fa\right)}^{2}{cos}^{2}(2\pi ft)}dt}\\ {+\mu k}_{1}a_{p}{}^{a_{1}}k_{2}a_{p}{}^{a_{2}}\\ F_{gr}{=F}_{grc}{+F}_{grf}=\frac{F_{0}a_{p}^{2r}{tan}^{r}\theta \cdot \left({u+a}_{p}tan\theta \right)a_{p}\int_{0}^{t}\sqrt{v_{w}{}^{2}{+v}_{a}^{2}{+2v}_{w}v_{a}cos(\omega t)}dt}{\int_{0}^{t}\sqrt{v_{w}^{2}{+v}_{s}{}^{2}{+2v}_{w}v_{s}cos\left[\omega t+\frac{b}{R}sin(2\pi ft+\varphi )\right]+{\left(2\pi fa\right)}^{2}{cos}^{2}(2\pi ft)}dt}\\ {+k}_{1}a_{p}{}^{a_{1}}k_{2}a_{p}{}^{a_{2}} \end{array}$$

From Equation (7), it can be seen that the grinding force of a single abrasive particle in LTUAG is related to the grinding depth, *a_p_*, grinding linear speed, *v_s_*, feed speed, *v_w_*, longitudinal ultrasonic amplitude, *a*, torsional ultrasonic amplitude, *b*, and longitudinal-torsional ultrasonic vibration frequency, *f*. During LTUAG, the grinding force increases with the increase of the grinding depth and the feed rate and decreases with the increase of ultrasonic amplitude, grinding linear velocity, and longitudinal-torsional ultrasonic vibration frequency. According to Equations (3) and (4), the grinding arc length of a single abrasive particle in LTUAG is longer than that of a single abrasive particle in ordinary grinding. The grinding force in LTUAG is smaller than that in ordinary grinding under the same grinding conditions.

### 2.3. Establishment of an Abrasive Particle Surface Temperature Model for TC4 Titanium Alloy in LTUAG

During the LTUAG of TC4 titanium alloy, intense friction is generated between the surface of the abrasive grain and the workpiece, and a large amount of frictional heat is generated, causing the temperature of the workpiece surface and the surface of the abrasive grain to rise, which in turn generates a temperature field on the surface of the abrasive grain and the workpiece, which eventually causes the thermal stress of the abrasive grain to fragment.

**Figure 3:**
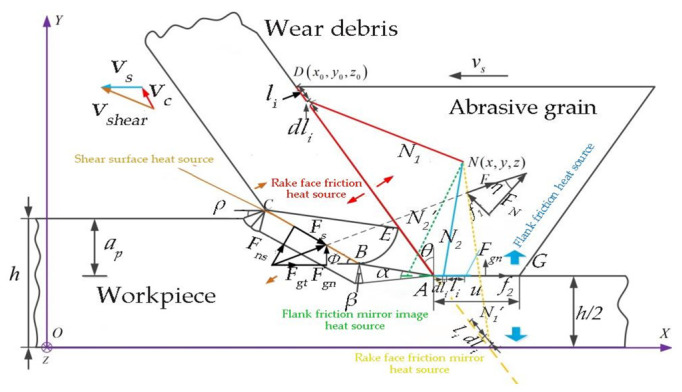
Model of surface temperature rise of a single abrasive particle in LTUAG.

Figure 3 shows the surface temperature rise model of a single abrasive particle during LTUAG of TC4 titanium alloy. According to Komanduri et al. [14,15], and assuming that the coordinate of a point in a single abrasive particle is N(*x*, *y*, *z*), then the temperature at the abrasive grain N(*x*, *y*, *z*) can be expressed as:

(8) $$T\left(x,y,z\right)=\frac{q_{m}}{{2\pi \lambda }_{t}}\int_{0}^{L^{t}}B(s)\int_{-\frac{w}{2}}^{\frac{w}{2}}\left(\frac{1}{R_{ts}}+\frac{1}{R_{ts}\prime }\right){dx}_{i}{dy}_{i}$$

where *λ_t_* is the tool’s thermal conductivity, *q_m_* is the heat source density, *L^t^* is the fixed heat source’s length, B(s) is the proportionality coefficient of grinding heat transfer into the grinding grain generated during grinding, *w* is the unit cutting width, *R_ts_* is the distance from the fixed heat source to a point inside the tool, and *R_ts_*′ is the distance from the mirror image heat source to a point inside the tool.

As can be seen from Figure 3, the heat that causes the temperature of the grinding grain to increase during the grinding process is mainly composed of two parts: one is the frictional heat generated by the relative sliding between the rake face of the abrasive particle and the chip, and the other is the frictional heat generated by the relative sliding between the bottom surface of the abrasive particle and the workpiece surface. The shear heat generated by the shear deformation of the workpiece material can only be transferred to the abrasive particle through the path of the shear surface heat source–chip–rake face of the abrasive particle, and the poor thermal conductivity of TC4 titanium alloy material leads to less heat transfer into the grinding grain. Therefore, this study does not consider the influence of the internal shear surface heat source of the workpiece on the surface temperature of the abrasive particles.

According to Figure 3, chip flow velocity, *v_c_*, and the length of the contact surface between abrasive particles and chips, *l_AD_*, can be expressed as:

(9) $$\{\begin{array}{l} v_{c}=\frac{v_{s}sin\phi }{cos\left(\theta +\phi \right)}\\ l_{AD}=\frac{a_{p}sin(\phi +\eta -\theta )}{sin\phi cos\eta } \end{array}$$

where *v_s_* is the grinding line speed, *ϕ* is the shear angle, *a_p_* is the grinding depth, and *η* is the friction angle.

According to Figure 3, combined with Equation (9), the friction heat source density on the rake face and the friction heat source density on the flank face of a single abrasive particle during LTUAG can be obtained (*q_f_*_1_, *q_f_*_2_) as:

(10) $$\{\begin{array}{l} q_{f1}=\frac{f_{1}\cdot v_{c}}{w\cdot l_{AD}}=\frac{\left(F_{gt}cos\phi {-F}_{gr}sin\phi \right)sin\left(2\eta \right){sin}^{2}\phi \cdot v_{s}}{w\cdot a_{p}sin\left[2\left(\phi +\eta -\theta \right)\right]cos(\theta +\phi )}\\ q_{f2}=\frac{f_{2}\cdot v_{s}}{w\cdot l_{AG}}=\frac{\mu \cdot F_{gr}\cdot v_{s}}{w\cdot u} \end{array}$$

where *f*_1_ is the friction force between the rake face of the abrasive particle and the chip interface, *f*_2_ is the friction force between the flank face of the abrasive particle and the workpiece surface, *w* is the unit cutting width, *l_AG_* is the length of the flank face of the abrasive grain, *l_AG_* = *u*, and *F_gt_* and *F_gr_* are the tangential grinding force and radial grinding force of a single abrasive particle in LTUAG. 

It can be seen from Equation (10) that the friction heat source density for the rake face and flank face of a single abrasive particle in LTUAG is related to the tangential grinding force, *F_gt_*, the radial grinding force, *F_gr_*, the grinding linear speed, *v_s_*, and the grinding depth, *a_p_*. The friction heat source density on the rake face of the abrasive grain in LTUAG increases with the increase of the tangential grinding force, *F_gt_*, radial grinding force, *F_gr_*, and grinding linear velocity, *v_s_*, and decreases with the increase of grinding depth, *a_p_*. The friction heat source density of the abrasive flank face increases with the increase of radial grinding force, *F_gr_*, and grinding linear velocity, *v_s_*, in LTUAG of a single abrasive particle.

During LTUAG, the heat generated by the friction heat source on the rake face of the abrasive particles is mainly transferred into the abrasive particles and chips, and the heat generated by the friction heat source on the flank face is mainly transferred into the abrasive particles and the workpiece. Therefore, the proportional coefficient, *B*(*s*) [16], of the grinding heat into the abrasive grains can be expressed by Equation (11):

(11) $$B(s)={\left(1+{0.974k}_{g}/\sqrt{r_{0}v_{s}K_{to}\rho c}\right)}^{-1}$$

Substituting Equations (10) and (11) into Equation (8), the temperature of the abrasive particle at N(*x, y, z)* in LTUAG can be obtained as:

(12) $$\begin{array}{c} T_{total}{=T}_{f1}{+T}_{f2}{+T}_{room}\\ =\frac{q_{f1}}{{2\pi \lambda }_{t}}\int_{0}^{l_{AD}}B\left(s\right)\int_{-\frac{w}{2}}^{\frac{w}{2}}\left(\frac{1}{N_{1}}+\frac{1}{N_{1}’}\right){dl}_{i}{dz}_{i}+\frac{q_{f2}}{{2\pi \lambda }_{t}}\int_{0}^{l_{AG}}B\left(s\right)\int_{-\frac{w}{2}}^{\frac{w}{2}}\left(\frac{1}{N_{2}}+\frac{1}{N_{2}’}\right){dl}_{i}{dz}_{i}{+T}_{room}\\ =\frac{{\left(\frac{1+{0.974k}_{g}}{\sqrt{r_{0}v_{s}K_{to}\rho c}}\right)}^{-1}}{{2\pi \lambda }_{t}}[\begin{array}{c} \int_{0}^{l_{AD}}q_{f1}\int_{-\frac{w}{2}}^{\frac{w}{2}}\left(\begin{array}{c} \frac{1}{\sqrt{{\left({x-x}_{0}{-l}_{i}sin\theta \right)}^{2}+{\left(y_{0}{-y-l}_{i}cos\theta \right)}^{2}+{\left({z-z}_{i}\right)}^{2}}}\\ +\frac{1}{\sqrt{{\left[l_{i}cos\left(\frac{\pi }{2}-\theta \right)\right]}^{2}{+y}^{2}+{\left({z-z}_{i}\right)}^{2}}} \end{array}\right){dl}_{i}{dz}_{i}\\ +\int_{0}^{l_{AG}}q_{f2}\int_{-\frac{w}{2}}^{\frac{w}{2}}\left(\begin{array}{c} \frac{1}{\sqrt{{\left({x-x}_{0}{-l}_{AD}{sin\theta -l}_{i}\right)}^{2}+{\left(y-\frac{t}{2}\right)}^{2}+{\left({Z-Z}_{i}\right)}^{2}}}\\ +\frac{1}{\sqrt{{\left({x-x}_{0}{-l}_{AD}{sin\theta +l}_{i}\right)}^{2}+{\left(y-\frac{t}{2}\right)}^{2}+{\left({z-z}_{i}\right)}^{2}}} \end{array}\right){dl}_{i}{dz}_{i} \end{array}]{+T}_{room} \end{array}$$

where *T_total_* is the average temperature inside the abrasive grain, *T_f_*_1_ is the temperature rise caused by the friction heat source of the abrasive grain’s rake face, *T_f_*_2_ is the temperature rise caused by the friction heat source of the abrasive grain’s flank face, and *T_room_* is the room temperature.

It can be seen from Equation (12) that the temperature at the abrasive particle N(*x*, *y*, *z*) during LTUAG is related to the grinding linear velocity, *v_s_*, and the friction heat source density of the abrasive grain rake face and flank face (*q_f_*_1_, *q_f_*_2_). During the grinding process, the surface temperature of the abrasive grains increases with the increase of the grinding linear velocity, *v_s_*, and the friction heat source density on the rake face and the flank face of the abrasive grains. In addition, according to Equations (7) and (10), the introduction of longitudinal-torsional ultrasonic vibration makes the tangential grinding force and the radial grinding force of a single abrasive particle (*F_gt_*, *F_gr_*), and the friction heat source of the density of the rake face and flank face of the abrasive grain (*q_f_*_1_, *q_f_*_2_), decrease. To a certain extent, the surface temperature of the abrasive grains is reduced, thereby reducing the influence of the temperature field of the abrasive grain surface on the fatigue wear of the abrasive grains and improving the durability of the grinding wheel.

### 2.4. Establishment of a Grinding Wheel Wear Model in LTUAG for TC4 Titanium Alloy

Grinding wheel wear of titanium alloy is an extremely complex physicochemical process, and its grinding wheel wear mechanism is relatively diverse. According to the research of scholars [17], the grinding wheel wear mechanism of titanium alloy in the grinding process is mainly abrasive wear and adhesive wear. To more accurately reflect the change of grinding wheel wear of TC4 titanium alloy in the process of LTUAG, the abrasive wear and adhesion wear mechanisms should be comprehensively considered, and a corresponding grinding wheel wear model should be established.

According to the research of Rabinowicz et al. [18,19], the tool wear rate caused by abrasive wear during tool cutting can be expressed as:

(13) $$\frac{{dW}_{\mathrm{ab}br}}{dt}{=Gv}_{r}$$

where $\frac{dW_{abr}}{dt}$ is the tool wear rate caused by abrasion wear, *G* is the characteristic correlation coefficient of abrasive wear of CBN, and *v_r_* is the workpiece feed rate during cutting.

According to the research of Usui et al. [20], the tool wear rate caused by the adhesive wear of workpiece material in the cutting process can be expressed as:

(14) $$\frac{{dW}_{adh}}{dt}{=A}_{w}v_{n}p_{n}exp\left(\frac{{-B}_{w}}{T_{m}}\right)$$

where *A_w_* and *B_w_* are the characteristic correlation coefficients of adhesive wear of CBN, *V_n_* is the relative sliding velocity of the chip contact surface, *P_n_* is the positive pressure of the contact surface between the tool and the workpiece, and *T_m_* is the tool surface temperature during cutting.

According to Figure 3, the positive pressure, *F_N_*, of the abrasive particle face during LTUAG can be expressed as:

(15) $$F_{N}=\frac{\left(F_{gt}cos\phi {-F}_{gr}sin\phi \right)cos\eta }{cos(\eta -\theta +\phi )}$$

As the abrasive particle will be chunk-broken, fall off, and binder-broken in both the initial wear stage and the rapid wear stage [21], it has a large impact on wheel wear, while the abrasive particle in the stable wear stage is in the form of abrasive wear and debris adhesion wear, and the abrasive wear rate changes are more stable. Assuming the grinding wheel enters the stable wear stage, the abrasive debris that is not discharged in time gradually fills the entire abrasive grain gap and adheres to the bottom surface of the abrasive grains participating in the grinding, thereby increasing the grinding force and grinding heat of the abrasive grain surface. The abrasive particles begin to break up and wear away. At this time, the abrasive particle breakage caused by the adhesion of abrasive debris is similar to the adhesive wear of the tool.

Substituting Equations (9) and (15) into Equation (14), and adding them to Equation (13) to obtain the grinding wheel wear rate, *r_wheel_(t),* of the stable wear stage in LTUAG can be expressed as:

(16) $$\begin{array}{l} r_{wheel}\left(t\right)=\frac{{dW}_{wheel}}{dt}=\frac{{dW}_{abr}}{dt}+\frac{{dW}_{adh\left(ch-t\right)}}{dt}+\frac{{dW}_{adh\left(t-w\right)}}{dt}\\ {=Gv}_{w}+\left[A_{w}p_{1}v_{c}exp\left(\frac{{-B}_{w}}{T_{f1}}\right){+A}_{w}p_{2}v_{w}exp\left(\frac{{-B}_{w}}{T_{f2}}\right)\right]\\ {=Gv}_{w}{+A}_{w}\left[\frac{\left(F_{gt}cos\phi {-F}_{gr}sin\phi \right)sin\phi cos\eta }{cos(\phi +\eta -\theta )\cdot cos(\theta +\phi {)v}_{s}}exp(\frac{{-B}_{w}}{T_{f1}}{)+F}_{gr}v_{w}exp(\frac{{-B}_{w}}{T_{f2}})\right] \end{array}$$

where $\frac{dW_{adh(ch-t)}}{dt}$ is the bonded wear rate at the interface between abrasive particles and chips, $\frac{dW_{adh(t-w)}}{dt}$ is the bond wear rate between the abrasive bottom surface and the workpiece contact surface, and *t* is the cutting time.

It can be seen from Equation (16) that the wear rate of the grinding wheel during LTUAG is related to the workpiece feed speed, *v_w_*, the grinding linear speed, *v_s_*, and the tangential grinding force and the radial grinding force of a single abrasive particle in LTUAG (*F_gt_*, *F_gr_*). The abrasive rake face temperature, *T_f_*_1_, and the abrasive flank temperature, *T_f_*_2_, are also related. It can be seen from Equations (7) and (12) that the grinding force of LTUAG and the surface temperature of abrasive grains increase with the increase of grinding depth and decrease with the increase of ultrasonic amplitude. Therefore, the grinding wheel wear rate of TC4 titanium alloy in LTUAG increases with the increase of the grinding depth and the workpiece feed rate and decreases with the increase of the grinding linear speed and the ultrasonic amplitude.

## 3. Verification and Analysis of the Grinding Wheel Wear Test of TC4 Titanium Alloy in LTUAG

### 3.1. Test Conditions and Program

**Figure 4:**
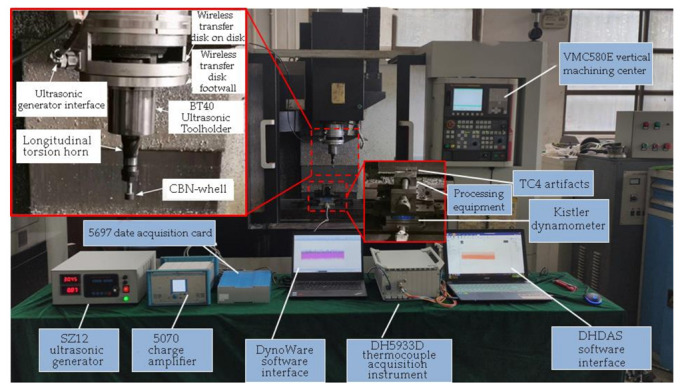
Test platform of TC4 titanium alloy in LTUAG.

The test platform of TC4 titanium alloy during LTUAG is shown in Figure 4. The test of TC4 titanium alloy during LTUAG was carried out on the VMC850E vertical machining center (Shenyang Shenyi Lathe Manufacturing Co., Ltd., Shenyang, China). The relevant parameters of the VMC850E vertical machining center are shown in Table 1. The longitudinal-torsional ultrasonic vibration system consisting of a wireless transmission device, an ultrasonic tool-holder, a longitudinal-torsional ultrasonic horn, and a ceramic CBN grinding wheel was installed on the spindle of the vertical machining center, and an external SZ12 intelligent ultrasonic generator (Hangzhou Jingzhen Ultrasonic Technology Co., Ltd., Hangzhou, China) to realize longitudinal ultrasonic vibration and torsional ultrasonic vibration of the grinding wheel. The relevant parameters of the SZ12 intelligent ultrasonic generator are shown in Table 2. After that, the grinding force and grinding temperature during the grinding process were collected by the Kistler 9257B dynamometer (Kistler Instrumente AG, Shanghai, China) and the DH5933D fast-response thermocouple acquisition instrument (Jiangsu Donghua Testing Technology Co., Ltd., Jingjiang, China), respectively.

**Figure 5:**
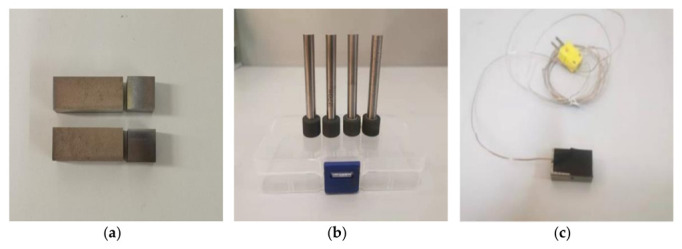
Workpieces, grinding wheels, and temperature test pieces used in the test. (**a**) TC4 workpiece. (**b**) Ceramic base CBN grinding wheel. (**c**) Temperature test piece.

In this experiment, a ceramic base CBN grinding wheel with a diameter of 15 mm and a particle size of 200# was used for grinding. The test piece used was TC4 titanium alloy (30 × 15 × 10 mm). The grinding wheel and workpiece are shown in Figure 5a,b, and the related information is shown in Table 3 and Table 4. The FA1004 precision electronic analytical balance (accuracy of 0.0001 g) produced by Zhejiang Lichen Technology Co., Ltd. (Shaoxing, China.) was used to detect the wear amount of the grinding wheel before and after the LTUAG test of TC4 titanium alloy. 

**Figure 6:**
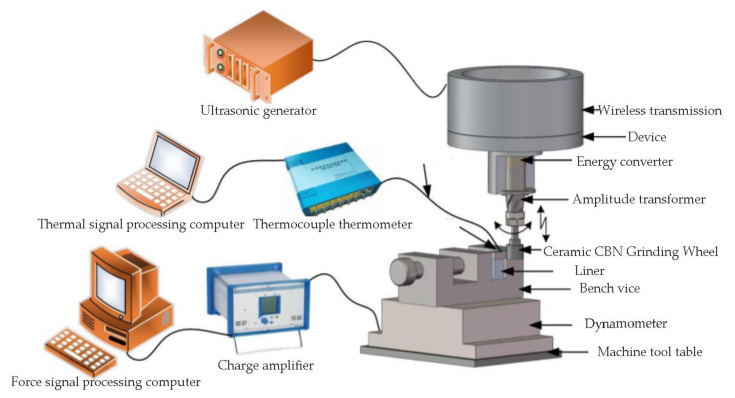
The working principle of force and thermocouples.

The temperature measurement specimen equipped with the thermocouple sensor is shown in Figure 5c. The whole measurement process was as follows: Firstly, a through hole with a diameter of 1 mm was machined by the CNC milling machine in the TC4 titanium alloy workpiece. Secondly, the K-type thermocouple sensor was put into the through hole, and the thermocouple tip was placed 0.3 mm away from the workpiece surface to ensure that the grinding wheel surface is in contact with the thermocouple tip during the grinding process, which can improve the accuracy of the measurement results. Thirdly, the thermocouple sensor and workpiece were tightly wrapped with insulating tape, the working end of the thermocouple sensor was fixed near the machining area of the workpiece, and the sensor output was connected to the thermocouple collector; then, the grinding temperature measurement system was built. The working principle of the thermocouple acquisition instrument is shown in Figure 6.

To analyze the influence of grinding process parameters and longitudinal-torsional ultrasonic parameters on the grinding force, grinding temperature, and grinding wheel wear rate in the process of LTUAG of TC4 titanium alloy, in this study, the longitudinal-torsional ultrasonic single-factor grinding test of TC4 titanium alloy was carried out. The parameters of the single-factor grinding test are shown in Table 5.

### 3.2. Test Results and Analysis

#### 3.2.1. Influence of Process Parameters on Grinding Force in LTUAG

**Figure 7:**
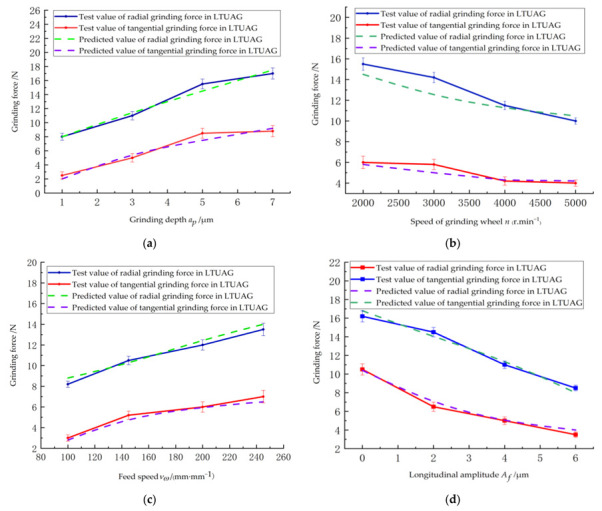
Influence of process parameters on the grinding force of LTUAG. (**a**) Depth of grinding. (**b**) Speed of the grinding wheel. (**c**) Feed rate. (**d**) Lengthening amplitude.

Figure 7 shows the variation of grinding force under different parameters during LTUAG. Figure 7a shows that the grinding force during LTUAG increased with the grinding depth. The radial grinding force and tangential grinding force showed an increasing trend during LTUAG when the grinding depth exceeded 5 μm, and the trend gradually slowed down. The volume of material removed increased with the grinding depth, which resulted in an increase of the grinding force. When the grinding depth exceeded 5 μm, the temperature induced by the grinding was higher than the recrystallization temperature of the workpiece, and the microhardness of the workpiece was lower than the original microhardness of the workpiece. As a result, when the grinding depth was further increased, the increasing trend of the grinding force became slower. 

Figure 7b shows that the grinding force during LTUAG decreased as the grinding wheel speed increased. The number of abrasive grains and the grinding arc length of the abrasive grains also increased as the speed of the grinding wheel increased, which in turn reduced the cross-sectional area of abrasive debris and the degree of adhesion wear of the single abrasive particle. Ultimately, the grinding force during LTUAG was reduced.

Figure 7c shows that the grinding force during LTUAG generally increased as the workpiece feed rate increased. With the increase of the feed rate, the volume of the workpiece material removed by the abrasive particles during the grinding process increased, and the deformation resistance of the workpiece material to the grinding wheel also increased, which led to an increase in the grinding force.

Figure 7d shows that the grinding force during LTUAG decreased as the longitudinal ultrasonic amplitude increased. As the longitudinal ultrasonic amplitude increased, the grinding arc length of the single abrasive particle lengthened, the cross-sectional area of the material removed decreased, and the chip deformation force decreased. The friction between the bottom surface of the abrasive grains and the workpiece was reduced, resulting in a reduction in the grinding force.

The experimental results of the grinding force under different process parameters during LTUAG are consistent with the changing trend of the theoretical prediction results of the grinding force during LTUAG, which verifies the accuracy of the grinding force model of LTUAG established in this study, as shown in Figure 7a–d.

#### 3.2.2. Effect of Process Parameters on Grinding Temperature of LTUAG

**Figure 8:**
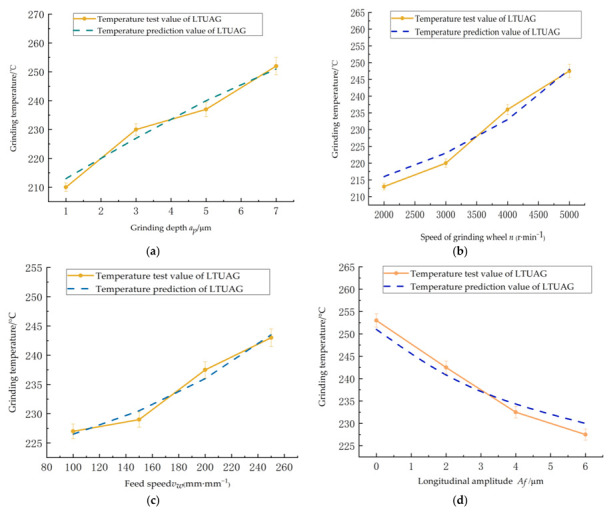
The influence of process parameters on the grinding temperature of LTUAG. (**a**) Depth of grinding. (**b**) Speed of the grinding wheel. (**c**) Feed rate. (**d**) Longitudinal amplitude.

Figure 8 shows the temperature variation of LTUAG under different process parameters. Figure 8a shows that the grinding temperature of LTUAG gradually increased as the grinding depth increased. The grinding force required to remove the workpiece material during LTUAG also increased as the grinding depth increased. This in turn led to the increase of the frictional heat source density on the contact surface of the abrasive grain and the workpiece as well as the increase of the grinding temperature. At the same time, the increase in grinding depth made the contact between the abrasive grain and the workpiece closer. The grinding area was more fully affected by the grinding heat and was not easily dissipated, which further accelerated the rising trend of the grinding temperature.

Figure 8b shows that the grinding temperature of LTUAG gradually increased as the grinding wheel speed increased, and the temperature of LTUAG started to rise faster when the grinding wheel speed exceeded 4000 r/min. This is because the friction heat source density between the abrasive particles and the workpiece increased as the grinding wheel speed increased, which in turn led to an increase in the grinding temperature. The vibration characteristics of LTUAG were weakened when the rotational speed of the grinding wheel exceeded 4000 r/min, which in turn reduced the heat dissipation rate of grinding, resulting in a faster rise in the grinding temperature.

Figure 8c shows that the grinding temperature of LTUAG gradually rose as the workpiece feed rate increased. This is because with the increase of the workpiece feed rate, the volume of TC4 material removed by the abrasive particles in LTUAG increased in unit time, which in turn led to an increase in the grinding force of LTUAG. The friction heat source density increased on the contact surface between the abrasive particles and the workpiece, and the grinding temperature increased. 

Figure 8d shows that as the longitudinal-torsional ultrasonic amplitude increased, the grinding temperature of LTUAG decreased overall. This is due to the increase in the longitudinal-ultrasonic amplitude and the decrease in the grinding force generated by the shear deformation of the workpiece material and the ploughing slip. In turn, the friction heat source density of the contact surface between the abrasive particles and the workpiece increased. At the same time, the contact-separation characteristics of the abrasive particles on the surface of the grinding wheel and the workpiece were strengthened, and the speed of grinding heat dissipation was accelerated. This led to a significant reduction in the temperature of the grinding area.

As shown in Figure 8a–d, the experimental results of the grinding temperature under different process parameters during LTUAG are consistent with the changing trend of the theoretical prediction results of the grinding temperature of LTUAG, confirming the accuracy of the grinding temperature model in LTUAG established in this study.

#### 3.2.3. The Effect of Process Parameters on the Wear Rate of Grinding Wheels in LTUAG

**Figure 9:**
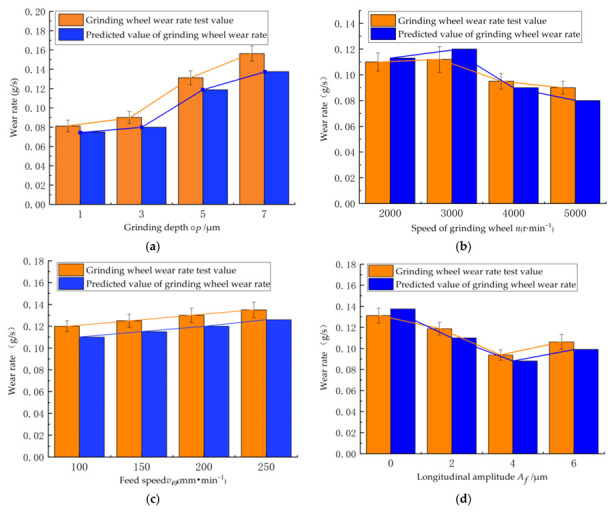
Influence of process parameters on grinding wheel wear rate in LTUAG. (**a**) Depth of grinding. (**b**) Speed of the grinding wheel. (**c**) Feed rate. (**d**) Lengthening amplitude.

A single-factor test on the grinding wheel wear rate of TC4 titanium alloy was carried out according to the single-factor grinding test scheme shown in Table 5, and the results of the single-factor test are shown in Table 6. The variation of the grinding wheel wear rate for LTUAG with different process parameters is shown in Figure 9.

Figure 9a shows that as the grinding depth increased, the wear rate of the grinding wheel under LTUAG increased overall. This is because with the increase in the grinding depth, the grinding force and grinding temperature also increased, which led to an increase in stress on the surface of abrasive particles caused by the coupling of mechanical stress and thermal stress. The crushing degree of abrasive particles was intensified, and the wear rate of the grinding wheel showed an increasing trend.

Figure 9b shows that with the increase of the grinding wheel speed, the grinding wheel wear during LTUAG increased and then decreased. This is because with the increase of the grinding wheel speed, the relative sliding speed of the contact surface between the abrasive grain and the workpiece increased, which led to an increase in the temperature of the front and flank surfaces of the abrasive grain, resulting in a gradual increase in the wear rate of the grinding wheel. When the grinding wheel speed exceeded 3000 r/min, the increase of grinding grains on the grinding wheel surface made the grinding force decrease, and the rising trend of grinding temperature slowed down, thus alleviating the grinding wheel wear to a certain extent, and the grinding wheel wear rate decreased.

Figure 9c shows that the wear rate of the grinding wheel under LTUAG increased as the workpiece feed speed increased. This is because as the feed speed increased, the contact pressure and temperature increased, which further intensified the degree of bond wear of abrasive particles. The wear amount caused by the bonding wear of the front and rear tool surfaces of the abrasive particles gradually increased.

Figure 9d shows that the grinding wheel wear rate decreased as the longitudinal ultrasonic amplitude increased and increased when the longitudinal ultrasonic amplitude exceeded 4 μm. This is because the grinding force and grinding heat during LTUAG were reduced as the longitudinal amplitude increased, which in turn led to the reduction of the wear rate. The excessively large, high ultrasonic amplitude increased the instantaneous high-frequency impact between the grinding wheel and the workpiece when the longitudinal amplitude exceeded 4 μm, which in turn led to an increase in the internal stress of the abrasive particles on the surface of the grinding wheel and the bonding agent. The degree of fragmentation of the abrasive particles and the bonding agent was increased, and the wear rate of the grinding wheel significantly increased. In addition, under the same processing conditions, the wear rate of the grinding wheel during LTUAG was reduced by 25.2% compared with ordinary grinding, indicating that LTUAG helped to improve the durability of the grinding wheel.

Figure 9a–d show that the experimental results of the grinding wheel wear rate during LTUAG under different process parameters agree with the theoretical prediction results of the grinding wheel wear rate of LTUAG. The changing trend was consistent, which verified the accuracy of the grinding wheel wear model during LTUAG established in this study.

## 4. Conclusions

1. The grinding force model and the grinding abrasive surface temperature model of LTUAG were established based on the single-grain grinding arc length model in LTUAG, and on this basis, the grinding wheel wear model of LTUAG was established by combining the wear model and the bond wear model. The theoretical analysis results showed that the grinding wheel wear rate of LTUAG increased as the grinding depth and workpiece feed rate increased and decreased as the grinding linear speed and ultrasonic amplitude decreased.
2. The single-factor test of LTUAG of TC4 titanium alloy showed that the grinding force and grinding temperature increased as the grinding depth and workpiece feed rate increased and decreased as the longitudinal amplitude increased. The grinding force gradually decreased as the grinding wheel speed increased, and the grinding temperature gradually increased as the grinding wheel speed increased. In addition, the variation trend of the experimental results of the grinding force and grinding temperature under different process parameters was consistent with the theoretical prediction results, which verified the accuracy of the established grinding force model and grinding temperature model in LTUAG.
3. The single-factor test results of the grinding wheel wear rate of TC4 titanium alloy in LTUAG showed that the use of longitudinal-torsional ultrasonic vibration reduced the wear rate of the grinding wheel by 25.2%, thereby increasing the service life of the grinding wheel and improving the machining efficiency of TC4 titanium alloy. The experimental results of the grinding wheel wear rate were consistent with the theoretical prediction results during LTUAG, which verified the accuracy of the grinding wheel wear model in LTUAG established in this study.

## Figures and Tables

**Table 1:** The parameters of VMC850E machining center performance.

Project	Parameter
The maximum travel of the table (*x*-axis)	850 mm
The maximum travel of the table (*y*-axis)	500 mm
Maximum stroke of spindle (*z*-axis)	540 mm
Range of rotation	50~8000 r/min
Maximum spindle power	11 kW
Maximum output torque	35.8 N∙m
Positioning accuracy	0.005 mm

**Table 2:** Performance parameters of the SZ12 ultrasonic frequency generator.

Project	Parameter
Input voltage	AC voltage 220 V
Frequency	50 Hz
Output power	Greater than 250 W
Frequency adjustable range	18–23 KHz

**Table 3:** Grinding wheel specifications.

Project	Parameter
Grinding wheel model	1A1W-type ceramic base CBN flat grinding wheel
Manufacturer	Zhengzhou Abrasives Grinding Research Institute Co., Ltd.
Grinding wheel length/mm	10
Wheel diameter/mm	15
Grinding wheel particle size	100#, 200#, 300#
Grinding wheel concentration	100%

**Table 4:** Physical and mechanical properties of TC4 titanium alloy.

Elongation δ5 (%)	Coefficient of Thermal Conductivity	Density	Tensile Strength	Hardness
≥10	7.955 W/m·K	4.5 g/cm^3^	≥850 MPa	HRC30

**Table 5:** Longitudinal-torsional ultrasonic single-factor grinding parameters of TC4 titanium alloy.

Exp. Number	Grinding Depth (μm)	Speed of Grinding (Wheel r/min)	Feed Speed (mm/min)	Ultrasonic Amplitude (µm)
1	1/3/5/7	4000	150	4
2	3	2000/3000/4000/5000	150	4
3	3	4000	100/150/200/250	4
4	3	4000	150	0/2/4/6

**Table 6:** Single-factor experimental results of grinding wheel wear rate in LTUAG of TC4 titanium alloy.

Grinding Depth (µm)	Wheel Speed (r·min^−1^)	Feed Speed (mm·min^−1^)	Longitudinal Amplitude (µm)	Grinding Length (mm)	Wear Rate (g/s)
1	4000	150	4	150	0.0813
3	4000	150	4	150	0.0951
5	4000	150	4	150	0.1313
7	4000	150	4	150	0.1525
3	2000	150	4	150	0.1090
3	3000	150	4	150	0.1130
3	4000	150	44	150	0.0950
3	5000	150	4	150	0.0910
3	4000	100	4	150	0.1183
3	4000	150	4	150	0.1255
3	4000	200	4	150	0.1294
3	4000	250	4	150	0.1378
3	4000	150	0	150	0.1317
3	4000	150	2	150	0.1196
3	4000	150	4	150	0.0953
3	4000	150	6	150	0.1079

## Data Availability

Not applicable.
